# Role of Regulatory T Cells in Chronic Obstructive Pulmonary Disease

**DOI:** 10.1155/pm/5048054

**Published:** 2025-03-06

**Authors:** Meghashree Sampath, Geetanjali Bade, Randeep Guleria, Anant Mohan, Sudip Sen, Anjana Talwar

**Affiliations:** ^1^Department of Physiology, AIIMS, New Delhi, India; ^2^Department of Pulmonary, Critical Care and Sleep Medicine, AIIMS, New Delhi, India; ^3^Department of Biochemistry, AIIMS, New Delhi, India

**Keywords:** COPD, flow cytometry, induction, regulatory T cells, suppression assay, T cells

## Abstract

**Background:** Chronic obstructive pulmonary disease (COPD) is a progressive lung disorder characterized by poorly reversible airway obstruction. COPD being an inflammatory disorder has been proposed to have an imbalance between proinflammatory and anti-inflammatory factors. Regulatory T cells (Tregs) being a negative regulator of immune response have been observed to play an important role in other inflammatory diseases as well as animal models of inflammation.

**Objective**: This study is aimed at assessing the suppressive functions of circulatory Tregs and examining the inductive capacity of naive CD4+ T cells to generate induced Tregs.

**Methods:** The study was conducted in 20 COPD patients (smokers *n* = 10; reformed smokers *n* = 10) and 20 age-matched healthy controls (smokers *n* = 10; nonsmokers *n* = 10). Peripheral blood mononuclear cells were isolated from blood using Ficoll density gradient separation. The suppressive functions were evaluated by assessing the proliferation of T responder cells (CD4+CD25−) in the presence of circulatory Tregs (CD4+CD25+) under polyclonal stimulation. In addition, cytokine-mediated suppression was assessed in the culture supernatants of the suppression assay. Inductive capacity was assessed by stimulating naive CD4+ T cells to generate iTregs in the presence of anti-CD3, IL-2, and TGF-*β*1.

**Results:** The percent suppression of T responder cells by Tregs was significantly lower in COPD smokers (*p* = 0.03) and COPD reformed smokers (*p* = 0.04) as compared to control smokers. On the assessment of cytokine-mediated suppression, significantly reduced IL-2 in COPD S as compared to COPD RS (*p* < 0.05) and reduced IL-10 and TGFß1 in COPD S as compared to CNS (*p* < 0.05) and CS (*p* < 0.05) was observed in the culture supernatants of suppression assay. In addition, a significantly higher frequency of iTregs with phenotype CD4+CD25+CD45RA+CD127− was observed in COPD S as compared to COPD RS (*p* < 0.01).

**Discussion:** Characteristics changes were observed in patients with COPD. The compromised Tregs function, despite the increase in systemic inflammation, suggests a potential role of these cells in the pathogenesis of the disease.

**Summary**

• Regulatory T cells (Tregs) being negative regulators of immune response are of paramount importance for the induction and maintenance of peripheral tolerance.
• Our data point towards compromised Tregs function, despite the increase in systemic inflammation, suggests a potential role of these cells in the pathogenesis of chronic obstructive pulmonary disease (COPD).

## 1. Introduction

COPD is characterized by slowly progressive and irreversible damage to the airways caused by a significant exposure to noxious particles and/or gases. It is one of the major causes of morbidity and mortality worldwide [1].

COPD encompasses chronic obstructive bronchiolitis with small airway obstruction, emphysema with destruction of lung parenchyma and enlargement of air spaces, small airway closure, and loss of lung elasticity [2]. Although it has been postulated that COPD develops as a result of an exaggerated inflammatory response of the lungs to cigarette smoke, which is considered to be a major risk factor, that accounts for about 80% of the cases; however, the primary mechanism responsible for smoke-induced COPD pathogenesis, remains unclear. In addition, it is intriguing to know that the airway inflammation in these patients persists even after smoking cessation [3, 4].

Tregs being the negative regulators of immune response are of paramount importance for the induction and maintenance of peripheral tolerance and they play a vital role in combating excessive immune response and autoimmunity. These cells have the ability to suppress the expression of activation markers, cytokine production, and proliferation in a diverse range of immune cells from both the innate and adaptive immune systems [5].

To the best of our knowledge, the literature review revealed discrepancies in the relative numbers and functional capacity of peripheral Tregs (pTregs) as well as a paucity of reports regarding the inductive capacity of naïve CD4+ T cells to generate induced Tregs (iTregs). Therefore, the current study is aimed at understanding the comprehensive role of Tregs in patients with COPD which might aid in gaining better knowledge about the pathophysiological mechanisms crucial for initiation and progression of the disease.

## 2. Methods

### 2.1. Participants

COPD patients were recruited after prior diagnosis by clinicians in the Department of Pulmonary, Critical Care and Sleep Medicine, AIIMS, New Delhi, India. Written informed consent in accordance with the 2013 amendment of the Helsinki Declaration was taken from all participants before being enrolled in the study. The study was approved by the Institute ethical committee (IECPG-115/26.04.2017, RT-09/2017 dated 14.08.2017) for human subjects. Participants were divided into two groups, patients and controls, and each group was further subdivided into COPD smokers (COPD S) (at least 10 pack years), COPD reformed smokers (COPD RS) (quit smoking for at least a year), age-matched control nonsmokers (CNS) (< 10 cigarettes in a lifetime), and control smokers (CS) (at least 10 pack years). Clinically diagnosed, stable COPD patients (male) between the ages of 30 and 70 years and in Stages II–III (moderate to severe) of the disease were recruited. All patients with a history of respiratory conditions other than COPD, any active inflammatory disease, exacerbations, or infections within the previous month were excluded.

### 2.2. Spirometry

A spirometry manoeuvre was carried out to measure the lung volumes and capacities. Forced vital capacity (FVC) is the maximum volume of air that can be expired forcibly after full inspiration; forced expiratory volume (FEV_1_) in the first second of the FVC manoeuvre and a ratio of FEV_1_ to FVC (FEV_1_ % FVC) were used to assess airway obstruction along with its severity in patients with COPD. A percentage of the predicted values was used to diagnose and categorise the patients according to the severity of the disease.

### 2.3. Collection of Human Peripheral Blood Samples

Following enrolment, 8 mL of peripheral blood was taken from controls and COPD patients. Blood was immediately transferred to an ethylenediamine tetraacetic acid (EDTA) vial for peripheral blood mononuclear cells (PBMCs) isolation. For serum separation, samples were collected in a plain vial.

### 2.4. Sample Processing

PBMCs were isolated using the density gradient centrifugation technique with Ficoll at 1:1 ratio. Following isolation, cells were washed with phosphate-buffered saline (PBS), counted and divided for downstream processing. For serum separation, peripheral blood was allowed to clot for 30 min and centrifuged for 20 min at 1000 × g, aliquot stored at ≤ −20°C.

### 2.5. Naïve CD4+ T Cell Isolation

From PBMCs, naïve CD4+T cells were isolated by negative selection method, using MagCellect Human Naïve CD4+T Cell Isolation Kit (#Cat MAGH115, R&D). Isolated CD4+ T cells were then utilized for in vitro induction.

### 2.6. Induction of Isolated Naïve T Cells to Generate iTregs

For the coating of culture plates, a day before sample processing, six well culture plates (#Cat 0030720113, Eppendorf, United States) were coated with anti-CD3 antibody (#Cat 130-093-387, MACS) at a concentration of 2 *μ*g/mL diluted in sterile 1X PBS, pH 7.4 and stored at 4°C [6]. On the next day, culture plates were prewarmed at 37°C, PBS with anti-CD3 was aspirated, and wells were washed with 1X PBS. Isolated naïve CD4+ T cells were resuspended in complete media containing RPMI-1640 presupplemented with 2.06 mM Glutamax-I and 25 mM 4-(2-hydroxyethyl)-1-piperazineethanesulfonic acid (HEPES) buffer (#Cat 72400–047), 10% heat-inactivated fetal bovine serum (FBS) (#Cat RM10679, HiMedia), 100 U/mL penicillin, 100 ug/mL streptomycin (#Cat A004, HiMedia), 2 ng/mL TGF-*β* (#Cat 240-B, R&D), and 5 ng/mL IL-2 (#Cat 202-IL, R&D) at a concentration of 1 × 10^6^ cells/mL. Cells were cultured at 37°C with 5% CO_2_ for 5 days and were routinely examined for any kind of contamination under a microscope. Also, the wells were replaced with fresh complete media (TGF-*β*1 and IL-2) each day. The culture was then terminated on 5^th^ day for flow cytometry experiments.

### 2.7. Antibody Staining for Flow Cytometry

Optimisation of antibody staining was performed by antibody titration and appropriate controls including isotype staining and fluorescence minus one (FMO) controls. Cultured cells at the end of 5^th^ day were processed for flow cytometric staining. Titrated dilution of antibodies for Tregs (CD4-FITC (#Cat 317407, Biolegend), CD25-APC (#Cat 302609, Biolegend), CD45RA-PeCy7 (#Cat 304125, Biolegend), CD127-BV421 (#Cat 351309, Biolegend), were added and incubated in dark for 45 min at 4°C. The cells were washed twice with cold cell staining buffer and resuspended in 400 *μ*L of fixation buffer. Data was acquired on flow cytometer (LSR Fortessa X-20, BD Biosciences, United States).

Data was analysed using FLOWJO V10.1 (Tree Star, Ashland, Orlando, United States). During analysis, ungated events were initially plotted in forward scatter-area × side scatter-area (FSC-A × SSC-A) plots to identify morphologically similar cells, followed by forward scatter-area × forward scatter-height (FSC-A × FSC-H) plots for the selection of single cells and exclusion of cell aggregates located far from the main diagonal. These cells were then gated for cell surface markers depending on cell type.

### 2.8. Isolation of CD4+CD25+ Tregs From PBMCs

Tregs were isolated using CD4+CD25+ regulatory T cell kit (#Cat 130-091-301, MiltenyiBiotec) yielding T responder cells (CD4+CD25-) and Tregs (CD4+ CD25+) via a two-step process. Firstly, CD4+ T cells were isolated via negative selection. Magnetically labelled cells (non-CD4+ cells) were retained within the column, whereas the unlabelled cells passed through the column and this cell fraction was collected as CD4+ cells. After washing the flow through, CD25 microbeads were added, the unlabelled cells that passed through the column were collected (CD4+ CD25- T responder cells), and the column was washed with the appropriate amount of buffer. Then, the column was removed from the separator and placed on a suitable collection tube. An appropriate amount of buffer was pipetted onto the column and the magnetically labelled cells (CD4+ CD25+ Tregs) were immediately flushed by firmly pushing the plunger onto the column. Finally, the cell pellet was resuspended in 1 mL PBS supplemented with media and processed for suppression assay.

### 2.9. Treg Suppression Assay

T responder cells (CD4+CD25−) were stained with the cell trace violet dye (CellTrace violet cell proliferation kit (#Cat C34557, Invitrogen)), a fluorescent dye that gets serially diluted and allows cell division to be tracked by flow cytometry. Appropriate volumes of T responders (5 × 10^5^cells) and Tregs (5 × 10^5^cells) isolated from CD4+ CD25+ regulatory T cell isolation kit and Human Treg Suppression Inspector beads (#Cat 130-092-909, Miltenyi Biotec Ltd., United Kingdom) were added to round-bottom 96-well plate at 1:1 bead:lymphocyte ratio in each well to give a polyclonal (CD2, CD3, and CD28) stimulus for proliferation. With RPMI 1640 media + 10% FBS, the total amount of each well was adjusted to 210 *μ*L. This plate was then incubated for 5 days, at 37°C with 5% CO_2_. At the end of the 5^th^ day, cultured cells were removed from wells, stained with CD4-FITC, and run on a flow cytometer.

### 2.10. Secretory Profiling

Using the LEGENDplex human essential immune response panel (13-plex: 740930), secretory profiling was carried out (IL-4, IL-2, CXCL10 (IP-10), IL-1*β*, TNF-*α*, CCL2 (MCP-1), IL-17A, IL-6, IL-10, IFN-*γ*, IL-12p70, CXCL8 (IL-8), and free active TGF-*β*1). The assay flow cytometry standard (FCS) files were analysed using BioLegend's LEGEND plex data analysis software.

### 2.11. Statistical Analysis

The statistical analysis was performed using the Kruskal–Wallis testing with post hoc Dunn's multiple comparison test. *p* value < 0.05 was considered to be statistically significant. A single asterisk (⁣^∗^) indicates a *p* value < 0.05; two asterisks (⁣^∗∗^) represents a *p* value < 0.01.

## 3. Results

### 3.1. Inductive Capacity of Naïve CD4+ T Cells to Generate iTregs

To examine the inductive capacity, naïve CD4+ T cells were isolated from PBMCs, 10 COPD patients and 10 controls were recruited and demographic details of the participants have been depicted in Table 1. On spirometric analysis, FEV_1_ reflecting airway obstruction was found to be significantly reduced in COPD S as compared to COPD RS (*p* = 0.007) Table 2.

#### 3.1.1. Gating Strategy of iTregs

**Figure 1:**
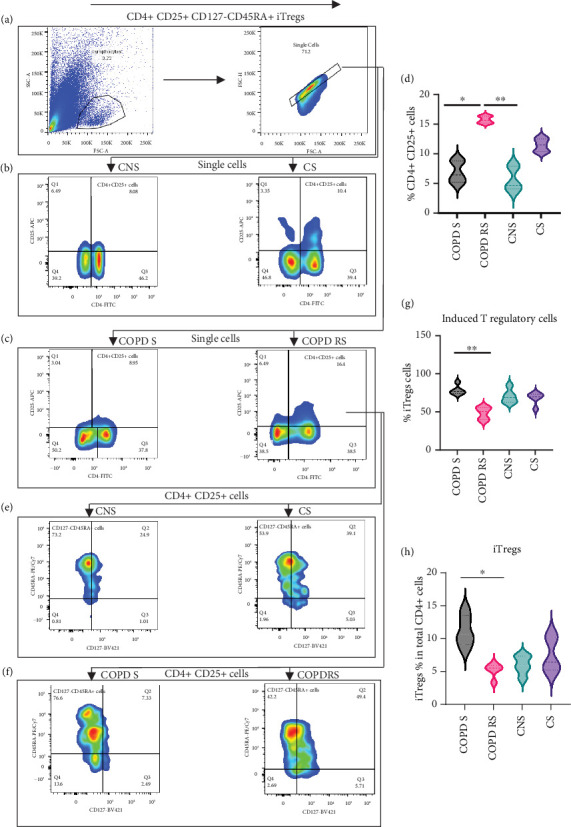
Representative pseudocolor plots showing sequential gating strategy used to identify induced Tregs. (a) Gating of single cells from lymphocytes. (b) CD4+CD25+ T cells in control nonsmoker (CNS), control smoker (CS), (c) COPD smoker (COPD S), and COPD reformed smoker (COPD RS). (d) Significantly higher frequency of CD4 + CD25+ T cells in COPD RS as compared to COPD S and CNS. (e) Gating of CD127-CD45RA+ from CD4+CD25+ cells in CNS, CS, (f) COPD S, and COPD RS. (g) Significantly lower frequency of iTregs in COPD RS as compared to COPD S. (h) Significantly lower frequency of iTregs as a percentage of total CD4+ T cells in COPD RS as compared to COPD S.

iTregs were identified by checking the expression of CD4, CD25, CD45RA, and CD127. Data has been represented as pseudocolor plots showing a sequential gating strategy (Figure 1). After gating for lymphocytes and single cells, cells were gated for the expression of CD4 and CD25. The dual positive cells (CD4+CD25+) were further gated for CD45RA and CD127. Subgroup analysis revealed a higher frequency of CD4+CD25+ T cells in COPD RS as compared to COPD S (*p* < 0.05) and CNS (*p* < 0.01). However, significantly higher frequency of iTregs with phenotype CD4+CD25+CD45RA+CD127− were observed in COPD S as compared to COPD RS (*p* < 0.01). In addition, analysis of iTregs as a percent of total CD4+ T cells also yielded similar results (*p* < 0.05).

### 3.2. Suppressive Functions of pTregs

To examine the suppressive functions, pTregs were isolated from PBMCs, for which another 10 COPD patients and 10 controls were recruited, and demographic details of the participants are depicted in Table 3. On spirometric analysis, FEV_1_ was found to be significantly reduced in COPD Ss as compared to COPD RS (*p* = 0.007) Table 4.

#### 3.2.1. Suppression Assay

**Figure 2:**
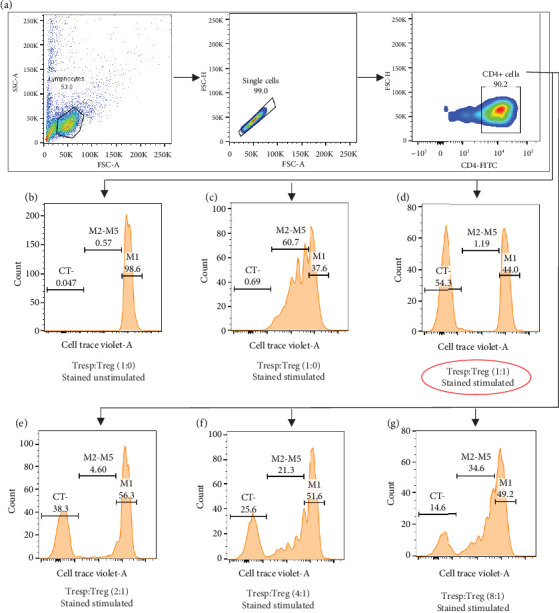
Representative pseudocolor plots and histograms showing sequential gating strategy used to identify the proliferation of T responders at different ratios of Tregs under polyclonal stimulus. (a) Gating of CD4+ cells from single cells and lymphocytes. (b–g) Proliferation of T responders in the presence and absence of Tregs. M1 indicates an undivided population, M2–M5 indicates different generations of daughter cells, and CT indicates (cell trace violet) negative population.

**Figure 3:**
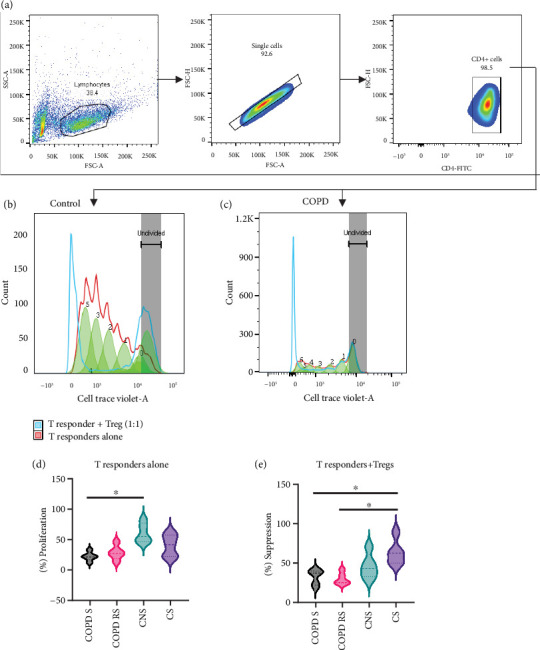
Representative pseudocolor plots and histograms show sequential gating of proliferation of CD4+ cells in the presence and absence of Tregs. (a) Gating of CD4+ cells from single cells and lymphocytes. (b, c) Overlaid histograms of T responders alone and T responder at 1:1 ratio with Tregs in controls and COPD, respectively. Violin box plots showing (d) significantly reduced proliferation in COPD smoker (COPD S) as compared to control nonsmoker (CNS), (e) significantly lower suppressive capacity of Tregs in COPD S and COPD reformed smoker (COPD RS) as compared to control smoker (CS). % suppression was calculated by 100 − (Tresp + Treg)/(Tresp alone)∗100.

Data has been represented as pseudocolor plots and histograms showing a sequential gating strategy used to identify the proliferation of T responders at different ratios of Tregs (Figure 2). After the gating of lymphocytes, single cells were gated, followed by CD4 expression. Further, these CD4+ cells were gated for cell trace violet dye. The stained-unstimulated cells were used for gating the undivided population and stained-stimulated cells were assessed to check the proliferation of T responder cells in the presence and absence of Tregs at 1:1 ratio. Percent suppression has been calculated by 100 − (Tresp + Treg, i.e., division with Tregs)/(Tresp alone, i.e., division without Tregs)∗100, and on comparison between subgroups (Figure 3), significantly lower suppressive capacity of pTregs was observed in COPD S (*p* < 0.05) and COPD RS (*p* < 0.05) as compared to CS. In addition, reduced proliferation (i.e., in the absence of Tregs) was also observed in COPD S as compared to CNS (*p* < 0.05).

#### 3.2.2. Secretory Profiling: Cytokine-Mediated Suppression by pTregs

**Figure 4:**
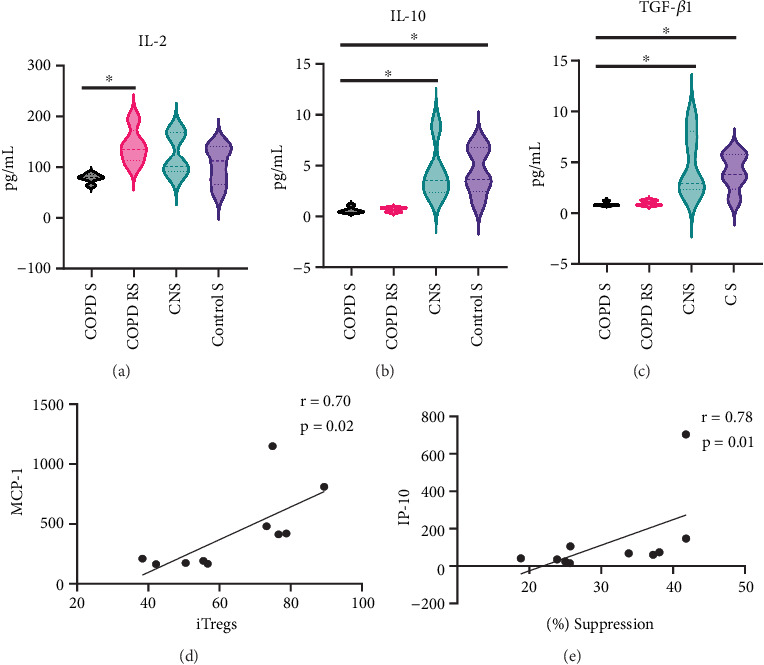
Violin box plots showing (a) reduced IL-2 in COPD S as compared to COPD RS. (b, c) Reduced IL-10 and TGFß1 in COPD S as compared to CNS and CS in culture supernatant of suppression assay. Correlation of induced Tregs, percent suppression, and serum cytokines. (d) Positive correlation of iTregs with MCP-1. (e) Positive correlation of percent suppression with IP-10.(COPD S, COPD smoker; COPD RS, COPD reformed smoker; CNS, control nonsmoker; CS, control smoker).

Culture supernatants of 10 COPD patients and 10 controls were analysed for the quantification of cytokines. Out of 13 cytokines assessed, only 3 cytokines were significantly different. Data has been represented as violin box plots showing reduced IL-2 in COPD S as compared to COPD RS (*p* < 0.05) and reduced IL-10 and TGFß1 in COPD S as compared to CNS (*p* < 0.05) and CS (*p* < 0.05) (Figure 4).

### 3.3. Correlation Between iTregs, Suppressive Capacity of pTregs, and Serum Cytokines

To explore the plausible association of serum cytokines on the inductive capacity of naïve CD4+ T cells and the suppressive capacity of pTregs, correlation analysis was performed. In the present study, a positive correlation was observed between MCP-1 with iTregs (*r* = 0.70) and IL-2 (*r* = 0.75). Suppressive capacity was also positively correlated with IP-10 (*r* = 0.78) (Figure 4); however, no significant correlation was found with spirometric parameters, smoking status, and clinical indicators of the disease.

## 4. Discussion

### 4.1. Suppressive Capacity of pTregs

Lower frequency of Tregs was observed in the peripheral blood of COPD patients along with decreased serum TGF ß1 levels [7] in previous experiments conducted in the lab, which necessitated the accurate assessment of the functional capacity of these cells to establish a strategy for the restoration of immunological homeostasis. Foxp3 is one of many markers that is expressed on activated effector T cells as well, especially at inflammatory areas, which could have been considered as Treg cells, highlighting the need for functional assays [8].

To the best of our knowledge, suppression assay using T responder and Treg coculture has not been studied so far in the peripheral blood of COPD patients; however, studies using other techniques have reported functional impairment. Experiments conducted by Lee et al. revealed decreased secretion of IL-10 from lung tissues of emphysema patients compared to controls, suggesting impaired pulmonary Treg function [9]. Also, the suppressive capacity of pulmonary Tregs from patients with lung fibrosis was decreased when compared to controls [10], supporting the theory that less potent Tregs are present in inflammatory lung disorders [11]. In contrast, Tregs inhibited autologous antigen-stimulated effector T cell proliferation from both COPD and controls [12]. However, in addition to being suppressed by Tregs in COPD patients, the authors observed exhaustive effector T cell phenotype (upregulation of surface PD-1) and altered CD4+CD25high CD127− after in vitro treatments [12] which might limit their capacity to perform effective antiviral and antibacterial effector functions [13].

The reduced suppressive capacity observed in the study points towards a disturbed immune homeostasis in patients with COPD. However, it is intriguing to note that the proliferative capacity of T responder cells was also reduced in the absence of Tregs, suggesting a potential exhaustive phenotype (with a decrease in serum IL-2) of the effector population which could be one of the reasons for the increased frequency of infections in patients with COPD. Foxp3+Tregs are phenotypically and functionally heterogeneous and can be divided into CD25++CD45RA+resting Tregs, CD25+++CD45RA- activated Tregs, or CD25++CD45RA− inflammatory cytokine–secreting T-cells [14, 15]. The cytokine-secreting T cells produce IFN*γ* and IL-17 have the least suppressive effect and are more prevalent in COPD patients [16]. The impaired Treg inhibition in COPD patients observed in the present study could also be due to the presence of nonsuppressive Tregs. Also, in the present study, percent suppression was positively correlated with serum IP-10, implying its possible role in the decreased suppressive capacity of Tregs in COPD.

### 4.2. Cytokine-Mediated Suppression

In the present study, a significant decrease in IL-10 and TGFß1 was observed in COPD Ss as compared to CSs and nonsmokers, owing to the reduced suppressive capacity of pTregs in COPD patients. IL-2 deprivation–mediated apoptosis has been reported to be one of the mechanisms of Treg suppression, where these cells consume IL-2 due to their inherent expression of CD25 and leave effector cells without the crucial cytokine, resulting in metabolic disruption and cell death [17, 18]. The significant decrease in IL-2 could be attributed to exhaustive effector T cells (T responders) along with impaired functionally suppressive Tregs in COPD Ss as compared to COPD RS. These findings, therefore, suggest a compromised Tregs function, via cell-cell contact as well as cytokine-mediated suppression in COPD patients.

### 4.3. Inductive Capacity of Naïve CD4+ T Cells to Generate iTregs

The relative numbers and suppressive functions were lower in the peripheral circulation of COPD patients. To examine, if the process of conversion of naïve CD4+ T cells to iTregs was compromised, the inductive capacity of naïve CD4+ T cell was assessed. The availability of an inadequate number of Tregs for in vivo research constitutes a limitation, given the small number of naturally occurring Tregs obtained by sorting peripheral blood cells as well as the poor in vitro expansion.

To the best of our knowledge, this is the first study to phenotypically assess the inductive capacity of naïve CD4+T cells to generate iTregs in COPD.

Previous studies have shown that stimulating murine CD4+CD25- cells through their T cell receptor (TCR) with antigen or anti-CD3 antibody in the presence of TGF*β*1 and exogenous IL-2 leads to the differentiation of CD4+CD25+ cells that expressed FOXP3 and were capable of inhibiting immune responses both in vitro and in vivo [19]. Similar results were observed in humans [20–22].

In the present study, a higher inductive capacity of naïve CD4+ T cells to generate iTregs under optimal stimulation with anti-CD3, IL-2, and TGFß1 was observed in COPD Ss as compared to reformed smokers. One might speculate that the persistent antigenic stimulation due to chronic noxious smoke exposure as well as elevated systemic inflammatory factors in COPD Ss might lead to certain characteristic changes in the naïve population of T cells which triggers the conversion into iTregs under optimal stimulation; however, this response apparently was not observable on smoking cessation in reformed smokers. In addition, iTregs were also found to be positively correlated with serum MCP-1, implying a plausible role of inflammatory mediators in the induction of naïve CD4+ T cells to generate iTregs in vitro.

## 5. Conclusion

In conclusion, our data point towards a disturbed immune homeostasis in COPD. Specifically, the compromised Tregs function, despite the increase in systemic inflammation, suggests a potential role of these cells in the pathogenesis of the disease.

## Figures and Tables

**Table 1:** Demographic data of the participants for induction of naïve CD4+ T cells to iTregs.

**Parameters**	**COPD S (** **n** = 5**)**	**COPD RS (** **n** = 5**)**	**CNS (** **n** = 5**)**	**CS (** **n** = 5**)**
Age (years)	62 (65–57)	54 (58–48)	61 (66–49)	51 (65–49)
Height (cm)	168 (171–162)	168 (170–166)	167 (171–163)	162 (168–157)
Weight (kg)	69 (74–61)	70 (76–55)	70 (80–60)	70 (84–60)
BMI (kg/m^2^)	24.9 (25.4–22.9)	24.22 (27.4–19.2)	25.1 (27.3–22.7)	28.4 (33.3–21.3)
CAT score (0–40)	26 (32.5–22.5)⁣^∗^	17 (20.5–13.5)	NA	NA
Smoking status	Smoker	Reformed smoker	Nonsmoker	Smoker
Smoking pack years	21.3 (22.3–16.9)	—	—	22.1 (24.4–19.5)

*Note:* Data has been expressed as median (IQR).

Abbreviations: BMI, body mass index; CAT, COPD assessment test; CNS, control nonsmoker; COPD RS, COPD reformed smoker; COPD S, COPD smoker; CS, control smoker; NA, not applicable.

⁣^∗^Significant difference between the groups.

**Table 2:** Spirometric data of the participants for induction of naïve CD4+ T cells to iTregs.

**Parameters**	**COPD S (** **n** = 5**)**	**COPD RS (** **n** = 5**)**	**CNS (** **n** = 5**)**	**CS (** **n** = 5**)**
FEV_1_ %pred	45 (49–38.2)	58 (66–54)	80.1 (82.5–78.6)	79.5 (81.6–80.5)
FVC %pred	65 (66–57)	65 (69–55)	81.3 (80.9–78)	80.6 (82.1–79.8)
FEV_1_/FVC	61 (66–58)	66 (75–60)	70.3 (72.1–69.8)	71.3 (74.5–70.6)

*Note:* Data has been expressed as median (IQR).

Abbreviations: BMI, body mass index; CAT, COPD assessment test; CNS, control nonsmoker; COPD RS, COPD reformed smoker; COPD S, COPD smoker; CS, control smoker; NA, not applicable.

**Table 3:** Demographic data of the participants for suppression assay and cytokine-mediated suppression.

**Parameters**	**COPD S (** **n** = 5**)**	**COPD RS (** **n** = 5**)**	**CNS (** **n** = 5**)**	**CS (** **n** = 5**)**
Age (years)	58 (63.5–50)	60 (63–58)	58 (62–51)	59 (69–57)
Height (cm)	165 (168–160)	163 (170–160)	171 (171.5–168)	168 (171–163.5)
Weight (kg)	73 (77.5–59.5)	58 (68–54)	75 (79–63)	60 (65–50)
BMI (kg/m^2^)	27.6 (28.5–21.9)	20.7 (25.1–19.9)	25.6 (27.1–22.2)	21.2 (23.5–17.7)
CAT score (0–40)	10 (17–5.5)⁣^∗^	19 (26–15.5)	NA	NA
Smoking status	Smoker	Reformed smoker	Nonsmoker	Smoker
Smoking pack years	22.2 (23.3–20.4)	—	—	20.1 (23.4–18.7)

*Note:* Data has been expressed as median (IQR).

Abbreviations: BMI, body mass index; CAT, COPD assessment test; CNS, control nonsmoker; COPD RS, COPD reformed smoker; COPD S, COPD smoker; CS, control smoker; NA, not applicable.

⁣^∗^Significant difference between the groups.

**Table 4:** Spirometric data of the participants for suppression assay and cytokine-mediated suppression.

**Parameters**	**COPD S (** **n** = 5**)**	**COPD RS (** **n** = 5**)**	**CNS (** **n** = 5**)**	**CS (** **n** = 5**)**
FEV_1_ %pred	45 (48.2–38.7)⁣^∗^	60 (66.5–55.1)	80.1 (81.4–79.4)	81.5 (82.8–78.5)
FVC %pred	65 (67.5–62)	69 (71–57.5)	81.4 (81.7–79.4)	82.6 (81.1–78.6)
FEV_1_/FVC	60 (66–53.5)	66 (75.7–60.5)	71.4 (73.8–70.4)	73.5 (72.5–69.4)

*Note:* Data has been expressed as median (IQR).

Abbreviations: BMI, body mass index; CAT, COPD assessment test; CNS, control nonsmoker; COPD RS, COPD reformed smoker; COPD S, COPD smoker; CS, control smoker; NA, not applicable.

⁣^∗^Significant difference between the groups.

## Data Availability

The data supporting this study's findings are available on request from the corresponding author. The data are not publicly available due to privacy or ethical restrictions.
